# Barriers to the conduct of randomised clinical trials within all disease areas

**DOI:** 10.1186/s13063-017-2099-9

**Published:** 2017-08-01

**Authors:** Snezana Djurisic, Ana Rath, Sabrina Gaber, Silvio Garattini, Vittorio Bertele, Sandra-Nadia Ngwabyt, Virginie Hivert, Edmund A. M. Neugebauer, Martine Laville, Michael Hiesmayr, Jacques Demotes-Mainard, Christine Kubiak, Janus C. Jakobsen, Christian Gluud

**Affiliations:** 1 Copenhagen Trial Unit, Centre for Clinical Intervention Research, Rigshospitalet, Copenhagen University Hospital, Copenhagen, Denmark; 2 Orphanet, Institut National de la Santé et de la Recherche Médicale (INSERM), Paris, France; 3 European Clinical Research Infrastructure Network (ECRIN), Paris, France; 4 IRCCS Istituto di Ricerche Farmacologiche Mario Negri, Milan, Italy; 5 EURORDIS - Rare Diseases Europe, Paris, France; 6 Brandenburg Medical School, Neuruppin, and Witten/Herdecke University, Witten, Germany; 7 Centre de Recherche en Nutrition Humaine Rhone-Alpes, Université de Lyon 1, Hospices Civils de Lyon, Groupement Hospitaler Sud, Pierre Benite, France; 8 Division of Cardiac, Thoracic, Vascular Anaesthesia and Intensive Care, Vienna General Hospital Währinger Gürtel, Vienna, Austria; 9 Department of Cardiology, Holbæk Hospital, Holbæk, Denmark

**Keywords:** Randomised clinical trials, Challenges, Barriers, Bottlenecks, Hindrances, Evidence based clinical practice, Evidence based medicine

## Abstract

**Background:**

Randomised clinical trials are key to advancing medical knowledge and to enhancing patient care, but major barriers to their conduct exist. The present paper presents some of these barriers.

**Methods:**

We performed systematic literature searches and internal European Clinical Research Infrastructure Network (ECRIN) communications during face-to-face meetings and telephone conferences from 2013 to 2017 within the context of the ECRIN Integrating Activity (ECRIN-IA) project.

**Results:**

The following barriers to randomised clinical trials were identified: inadequate knowledge of clinical research and trial methodology; lack of funding; excessive monitoring; restrictive privacy law and lack of transparency; complex regulatory requirements; and inadequate infrastructures. There is a need for more pragmatic randomised clinical trials conducted with low risks of systematic and random errors, and multinational cooperation is essential.

**Conclusions:**

The present paper presents major barriers to randomised clinical trials. It also underlines the value of using a pan-European-distributed infrastructure to help investigators overcome barriers for multi-country trials in any disease area.

**Electronic supplementary material:**

The online version of this article (doi:10.1186/s13063-017-2099-9) contains supplementary material, which is available to authorized users.

## Background

Randomised clinical trials (RCTs) are essential when evaluating the efficacy and safety of all interventions, exploring new indications for authorised drugs, and comparing the efficacy and safety of approved healthcare strategies [1–4]. Nonetheless, common barriers to the conduct of RCTs are widely recognised and discussed [5]. RCTs are limited by a growing complexity that increases labour, impedes speed, and multiplies costs. In fact, between 2007 and 2011, applications to run clinical trials in Europe experienced a marked decline by 25% [6]. The presently effective EU Clinical Trial Directive 2001/20/EC combined with the shrinking economy are believed to have contributed to the significant decrease. If the decline is to be reversed, new strategies for improving how RCTs are organised and conducted are warranted, and barriers for the conduct of RCTs should be identified as a first step.

In 2008, Duley and co-workers [5] identified major barriers to the conduct of RCTs: inadequate funding; overly complex regulations producing needlessly complex trial procedures; excessive monitoring; over-restrictive interpretation of privacy laws without evidence of subject benefit; and inadequate understanding of methodology (Table 1). Multinational collaboration seems important for clinical research, as it might improve research quality, maximise access to patients, and lead to faster results. Multinational collaboration also enables the sharing of medical and scientific expertise, tools, procedures, and costs; increases the applicability of research findings; reduces duplication; and enhances methodological standards [7–9]. The evidence from multinational trials can support enhanced health policy-making, optimal resource use, and improved patient care across borders [7, 8]. Despite the advantages of multi-country cooperation, just 3% of academic trials compared to 30% of industry trials are multinational [10].

**Table 1:** Major barriers to the conduct of randomised clinical trials

Duley and co-workers’ five major barriers to RCTs [5]	Comments	ECRIN’s eight major barriers to RCTs	Comments
Inadequate funding	Still highly relevant, but if a very substantial proportion of clinical research is considered wasted, it might not be the most prominent problem	Inadequate identification of the clinical research questions	Can only be based on systematic reviews of the literature. Added as a new barrier
Overly complex regulations producing needlessly complex trial procedures	Still highly relevant	Inadequate knowledge and understanding of clinical research	Too often results from observational studies are used as evidence for interventions where randomised clinical trials ought to have been conducted
Excessive monitoring	Still highly relevant	Inadequate knowledge and understanding of clinical trials	Too often when the randomised clinical trial design is chosen, it is not properly designed and conducted
Over-restrictive interpretation of privacy laws without evidence of subject benefit	Still highly relevant	Inadequate funding	Funding could be used more effectively by teaching investigators how to properly use the clinical research designs available
Inadequate understanding of methodology	Still highly relevant - the major problems have now been highlighted and brought to the forefront	Inadequate infrastructures	Added as a new barrier
		Overly complex regulation	There is a need to harmonise regulations of clinical trials on all interventions globally
		Excessive, non-focused monitoring	Should be assisted more through central monitoring in the future
		Too restrictive privacy and lack of transparency	Still highly relevant Lack of transparency added as new barrier

Major barriers as identified by Duley and co-workers in 2008 [5], and by the present European Clinical Research Infrastructures Network (ECRIN) panel in 2017. *RCT* randomised clinical trial

In continuation of the work by Duley and co-workers, the present paper reports the discussion among the methodology task force of the European Union Framework Programme 7 (FP7) ECRIN Integrating Activity (ECRIN-IA) project on the most pronounced barriers to the conduct of RCTs, irrespective of the type of intervention, condition, or disease in question.

The present paper also briefly presents three key areas of research, which each have specific barriers, and will benefit from multinational cooperation: rare diseases, medical devices, and nutrition.^1^

## Methods

**Fig. 1:**
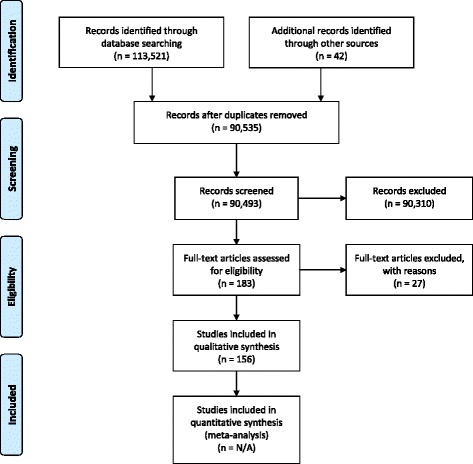
Preferred reporting items for systematic reviews and meta-analyses (PRISMA) 2009 flow diagram, depicting the process for selection of relevant literature

The approach for the present paper is based in part on systematic literature searches for appropriate articles using the following databases: The Cochrane Library (Wiley) (Issue 5 of 12, 2016) (including the Cochrane Database of Systematic Reviews (CDSR), CENTRAL, National Health Service Economic Evaluation Database (NHSEED), and Database of Abstracts of Reviews of Effects (DARE, US Library of Medicine)); MEDLINE (Ovid SP) (1946 to May 2016); EMBASE (Ovid SP) (1974 to May 2016); and Science Citation Index Expanded (1900 to May 2016), and search term combinations: “evidence* and (medicine or practice)) or (clinical trial*) or (systematic review*)” plus “barrier* or bottle*neck* or obstacle*”. Articles were selected if they included valid considerations of how barriers to the conduct of RCTs could affect their number, feasibility, and quality. The exact search strategy is provided in Additional file 1. A PRISMA flow diagram depicting the selection process and a preferred reporting items for systematic reviews and meta-analyses (PRISMA) checklist are provided in Fig. 1 and Additional file 2. These documents served as the basis for face-to-face and telephone discussions among the ECRIN-IA methodology task-force from 2013 to 2017.

## Results and discussion

### Search results

Through the electronic literature searches we identified a total of 90,493 references after removal of duplicates. The screening process narrowed the search down to 156 relevant references, which are listed in Additional file 3.

### General barriers to randomised clinical trials within all disease areas

Many challenges are shared by RCTs conducted in any disease area, but some may be disease-specific as discussed in more detail in other papers on rare diseases, medical devices, and nutrition [11–13]. The present paper focuses on the major barriers for all types of RCT.

#### Inadequate knowledge and understanding of clinical research and trial methodology

Selecting the right study design and methodology for the research question at hand is one of the major identified challenges [14, 15]. This is compounded by the tendency of some investigators to demonstrate the validity of an intervention (diagnostic, prognostic, preventive, or therapeutic) rather than to challenge the hypothesis until strong evidence supports the contrary (falsification of hypotheses). Methodological flaws contribute to the waste in clinical research as described in a series of papers published in *The Lancet* [16–22] where authors claim that 85% of biomedical clinical research is wasted. The sources of waste include selection of lower-priority research questions, under-use of prior systematic reviews of control and experimental interventions during the planning of a trial, underreporting of trials and trial results, inadequate description of interventions or outcomes, and inadequate trial analysis or interpretation (in the light of systematic reviews) [16–22].

A well-designed and well-conducted RCT is considered the gold standard in clinical research. RCTs rank top of the evidence hierarchy compared to other research approaches, as they employ the design least affected by bias, surpassed only by a systematic review of combinable, well-conducted RCTs [3, 4, 23–25]. But like any type of research, RCTs have their advantages and disadvantages, and can be subject to abuse. The key is not only to conduct more RCTs, but also to conduct trials that are substantially better, in terms of methodological quality, than the ones being conducted now. Evidence for the effectiveness of interventions should rely on well-conducted RCTs, but they are immensely expensive to conduct, and require time and resources rarely accessible to independent investigators. Due to the significantly lower cost and labour associated with observational studies (e.g., non-randomised, cohort, and case-control studies) these often seem an appealing alternative. Compared to RCTs, observational studies produce results that have less evidential weight [3, 4, 23–25], and even when they are large-scale and well-conducted, observational studies will often greatly overestimate or underestimate potential effects, and thus fail to determine the true balance between benefits and harms from an intervention [3, 4, 23, 26–29]. Accordingly, clinicians and investigators run large risks when they base treatment recommendations on observational evidence, especially when systematic reviews do not find an indication for the intervention at hand [30–34]. Observational studies are justified, however, when well-conducted and when assessing rare adverse events, late adverse effects, or long-term adverse effects [3, 35]. Well-conducted observational studies may also be important in designing a trial, providing information on anticipated incidences in the control groups, standard deviations when assessing continuous outcomes, and choice of baseline characteristics (predictors) for adjustment of analyses. Another justified role for well-conducted observational studies is in monitoring implementation of evidence-based interventions in clinical practice and documenting the natural history of diseases based on registries and databases [36].

#### Lack of funding

Clinical trials, and especially confirmatory RCTs, are both costly and time-consuming, and lack of funding remains one of the largest barriers towards their completion [37]. Large-scale clinical trials are often conducted at multiple sites, inevitably leading to a higher logistical and regulatory complexity, which is even more labour-intensive and cost-intensive. Moreover, many outcomes of interest occur after a significant time span, and therefore some interventions require long-term implementation and follow up.

Clinical research is typically funded either by industry or public entities. While pharmaceutical companies provide funding for development of a narrow pipeline of commercially attractive drugs, academic researchers often have to rely on public funding to conduct research they deem important to advancing science and medical practice [38]. Unfortunately, academic trials, which are independent and non-commercial, are often underfunded, making them too small and short-lived to provide high-quality evidence and reliable estimates of the long-term balance of risks and benefits [39]. In addition, decision makers and public funding bodies may restrict the use of funds beyond country borders, and beyond medical or health conditions about which little is known. Without high-quality academic trials, general public-health issues of limited interest for pharmaceutical companies may not be addressed.

From a global perspective, funding also affects the balance between the West and low-income and middle-income countries with regards to research resources [10]. More than a decade ago, the Global Forum for Health Research introduced the “10/90 gap”, a term used to explain that less than 10% of the world’s research resources were allocated for 90% of the health problems [40]. Concerns are that trials are initiated by the pharmaceutical and medical device companies principally for the benefit of the West [9, 41]. From an ethical view, clinical research should reflect the health needs and priorities in the countries where the research is conducted [42].

A major advancement in the funding of multinational, independent clinical trials is the European Commission Horizon 2020 (H2020) Research and Innovation programme, with nearly €80 billion of funding available over 7 years (2014 − 2020) [43]. Yet, H2020 is extremely competitive, allocating funding of up to 6 million Euros to only 20 trials per year [44]. The current acceptance rate under this programme is only 4% [44]. There are additional sources of European funding (e.g., E-Rare for rare-disease trials as of 2016; the Paediatric Clinical Research Infrastructure Network, or PedCRIN, for paediatric trials); however, current options are limited and cannot cover all European research needs.

#### Excessive monitoring

The International Conference on Harmonisation (ICH) is a standards organisation that provides good clinical practice (GCP) guidelines to protect the safety and rights of patients in trials [45]. The ICH-GCP guidelines are without legal power, but have been applied and adopted internationally [9, 45]. The ICH guidelines also describe monitoring obligations, which have been conjointly agreed by regulatory authorities and the industry, but curiously, without sufficient consultation with academic experts in trial methodology [46].

There are large logistical and financial burdens associated with frequent monitoring on site, which typically involve either monitoring of the clinical trial for quality and control, or conducting site education and training. Interestingly, there is no empiric evidence that supports the widespread adoption of ICH-GCP guidelines, and they have been criticised for their costly and time-consuming compliance activities without evidence of their usefulness [47, 48]. For certain trials, GCP trial monitoring may increase the costs of a trial by 30% to 40% or more [49].

#### Restrictive interpretation of privacy law and lack of transparency

Restrictive privacy laws impede the flow of health and private information that could help both researchers and healthcare personnel identify patients who might be offered enrolment into clinical trials. This is especially a concern when patients with rare conditions are difficult to recruit [12]. Privacy laws have become restrictive, in part due to the public’s lack of trust in the healthcare system regarding the handling of sensitive data. In a recent US study, the degree of mistrust was demonstrated when nearly 1/8 patients withheld health information from a healthcare professional during hospital admission [50].

Transparency refers to the degree of transparent information made publicly available on the authorisation, conduct, and results of a clinical trial [51]. Lack of transparency may come from failure to register and report clinical trials regardless of results [52, 53], but it could also come from inadequate descriptions of, e.g., procedures or adverse events, which in the public eye would only add to the dishonest reputation of commercial trials, and in the scientific eye would appear biased. Moreover, positive findings are more likely to be published compared to neutral results or harmful effects, consequently skewing research findings towards a more favourable outcome per stakeholder’s calculation. Such selective reporting is known as publication bias [38, 51, 52]. As clinical research essentially aims to provide high-quality evidence to help guide healthcare strategies appropriately, the hazard with publication bias and lack of transparency is that decision-making is based on faulty or incomplete information that may consequently bring harm to patients.

#### Overly complex or inadequate regulatory requirements

The Clinical Trials Directive from 2001 (2001/20/EC) commissioned by the EU, and currently in force, seeks to regulate and streamline clinical research across Europe [54–58]. Trial investigators and sponsors are required through this directive to ensure ethical review and authorisation by competent national authorities before enrolment into a trial, to manufacture drugs according to good manufacturing practice (GMP) principles, and to assure GCP standards during the conduct of a trial. Moreover, all trial-related changes are to be reported to the supervising authorities [58]. The restrictive nature of the directive means that each EU member must stray from national legislation and form a legal framework that will meet the imposed requirements, which has caused regulatory approval for clinical trials across Europe to become exceptionally complex [46, 55, 56, 58]. By increasing the administrative burden and time taken to launch new trials, the presently effective EU Clinical Trial Directive 2001/20/EC is believed to have contributed to the significant decrease in numbers of RCTs conducted in Europe, especially non-commercial RCTs [6, 59]. The European Commission recently developed a new Clinical Trial Regulation (EU No 536/2014), which aims to simplify procedures and harmonise regulatory requirements across Europe. The new regulation is scheduled to come into force no earlier than 2019 [60]. The ambition is to reverse the observed decline in RCTs, and to ensure that Europe remains an attractive site for clinical research [61].

The arguments for applying a high degree of complexity to regulatory requirements are unclear, and in some cases, the regulation is inadequate. Issues include: (a) approval by multiple ethics committees with different sets of requirements leading to multiple trial contracts, and moreover, a lack of well-educated ethics committees that may delay approval and regulatory assessments, e.g., by requiring excessive, explanatory details in the protocol and during participant enrolment [62, 63]; (b) multiple rules and different strategies for data management applied at different research sites or between countries that may lead to unnecessary costs and delays, and moreover, an increasing demand for reporting and storing information combined with inadequate data management systems, which could hamper data quality and thereby threaten the usefulness of results [64]; (c) pharmaceutical companies are not required to deliver placebo free of charge, which may hinder non-commercial research as valid placebos are expensive and cumbersome to produce. Accordingly, international laws should demand from manufacturers of drugs, device components, food additives, colour additives, or dietary supplements that get access to a market, to provide valid placebos for independent investigations of the product on request; (d) lengthy informed consent forms that include complex legal language, which, first, raises doubt about whether patients are truly informed about the trial, and second, may intimidate eligible participants, leading to poor accrual, delays, and premature termination of trials [58, 59]. It has even been claimed that requirements for consent forms focus on protecting the review board from risk rather than the participants [65]; and (e) complex safety reporting, including detailed recording of minor events (e.g., known adverse effects), and over-reporting of all serious adverse events to all relevant regulatory authorities, ethics committees, and site investigators, which could lead to an overwhelming bulk of reports with rarely useful insights or improved safety [58, 59, 66].

#### Inadequate infrastructures

Another barrier to the conduct of RCTs is insufficient development of research infrastructures to facilitate their development and management, especially when multiple countries are involved. Research infrastructures are specialised clinical research centres (CRCs) and academic clinical trial units (CTUs) that are organised into larger networks and offer services for the preparation, design, and conduct of clinical research for any disease area. Support can be provided, for example, on trial design and methodology; on the selection of appropriate outcomes; mapping of multinational investigation centres and clinical sites; development of the protocol (with independent scientific review); regulatory and ethical authorisations and follow up; and on-site monitoring, etc. [7]. However, research infrastructures are either completely missing or scarce in some countries, in particular the middle-income countries where public funding for clinical research is insufficient. And where they do exist, researchers may be unaware of them or neglect to make use of the tools, competencies, and expertise they offer. We suggest that the development and greater use of research infrastructures could help to improve RCTs (and other types of multinational clinical trials), particularly in regard to reporting and the scientific soundness of trials. Roughly 50% of all initiated clinical trials fail to complete their reporting [50–52], and given that many clinical trials are not found in registries or detailed information on them is unavailable, this number is likely an underestimation. Moreover, of all trials reported, major risks of systematic errors (bias), design errors, and random errors (play of chance) make the clinical value of the results highly questionable [17–23].

### Solutions to barriers to randomised clinical trials

This section presents potential solutions to the challenges detailed above, drawing on the experience of ECRIN-IA, applicable to clinical trials in any disease area, and in particular those involving multiple countries.

#### Training in clinical research and trial methodology

Lack of training and trial expertise at sites can affect overall site performance and recruitment [67]. In a 2013 ECRIN-IA survey on investigator needs conducting clinical trials on rare diseases, even experienced investigators pointed to a need for support in training their staff on both clinical research (37% of respondents) and in the conduct of multinational clinical trials (15% of respondents). Training courses could help to standardise and streamline definitions, data collection methods, and case report forms, and educate clinicians about rare diseases and non-previously reported or severe adverse events, among others [68]. This task will require concerted actions and investments by many stakeholders at local, regional, and national levels [68].

#### Funding options

One of the great challenges for independent academic trials is to reduce costs and to facilitate the successful pursuit of funding, in particular for multinational trials. The ECRIN-IA project provided funding through a competitive call using the “Transnational Access” scheme, whereby an investigator is eligible for free services offered by an infrastructure located in another country. Transposed in the field of clinical trials, this resulted in funding the extension of a trial outside the country of the principal investigator (and five multinational trials were supported according to this scheme). Another option to support multinational clinical trials is the combination of national public funding sources through an ERA-Net mechanism, as proposed in 2016 by E-Rare, or the combination of national charity funding. A new concept for independent funding, research crowdfunding, which extends beyond regions and country borders, was recently suggested [69, 70]. While this suggestion may seem far-stretched for some, raising funds in short time with aid from the public would provide clinical research with financial support and independency. Moreover, research crowdfunding invites the public to engage in the clinical research enterprise, which could be an incitement for greater transparency.

#### Simplifying monitoring

Regarding monitoring, the US Food and Drug Administration (FDA) and the European Medicines Agency (EMA) recently acknowledged the logistical and financial burden of excessive site visits by endorsing a regulation with a risk-based approach to monitoring. With this approach, the monitoring activities can be weighed and targeted according to needs [71–73]. However, the regulation only comprises drafts where the quality standard is set by the ICH-GCP guidelines, despite their intrinsic problems [48]. If a risk-based approach is to be implemented with success, it will require a comprehensive modification of the ICH-GCP guidelines. Monitoring should focus mainly on verifying that the safety and rights of patients are protected (e.g., consent procedures and reporting of serious adverse events), ensuring that the trial data are reliable (e.g., integrity of the randomisation and completeness of follow up), and on identifying important problems early on in each individual trial. A solution that was put forward as part of ECRIN-IA is centralised monitoring combined with a risk-based monitoring approach. This enables researchers to use resources more efficiently depending on the characteristics of the trial, without compromising the quality of data, in line with the Council Recommendation on the Governance of Clinical Trials (OECD) [74].

#### Increasing transparency

Restrictive privacy laws could be dealt with by addressing public trust in the healthcare system. One way this could be achieved is making information and results more transparent. In recent years, several international workforces, journals, and institutions have taken initiatives to promote transparency, and provide access to the huge amounts of existing data in the healthcare system, and other registries otherwise inaccessible to the public. Retrieving these data will allow researchers to collect all information available regardless of trial results. Transparency includes publication of detailed trial protocols published before trial launch and thorough reporting of all trial results [34, 51]. However, the usefulness of increased transparency is dependent on the research community and industry to submit accurate and informative data or information.

ECRIN-IA has worked to promote transparency in various ways [51]. As for all of the projects that ECRIN is involved in, ECRIN-IA committed to registering trials in a public register before inclusion of the first participant, publishing results irrespective of findings, and making raw anonymised data sets available to the scientific community upon request to the sponsor or principal investigator one year after the trial is completed (last follow up of the last patient) or, for registration trials, when registration is completed or the development is discontinued.

#### Dealing with complex ethical and regulatory processes

To simplify and speed up ethical and regulatory approvals, without compromising patient rights and the scientific validity of clinical trials, various solutions have been or could be implemented [49]. In Europe, the European Commission has already introduced centralised procedures for regulatory approval [9, 49]. However, this has not resolved all issues related to national regulatory and ethical approval, and there should be a more critical view of the measures taken by the European Commission to simplify ethical and regulatory approval. If a unique ethical approval is foreseen in the EU or in its single member states, the expertise involved in the evaluating boards should be adequate to the task. Also, there should be no reason to separate scientific and ethical assessment they cannot leave aside each other. There are parties that may be reluctant to support implementation of centralised, risk-based approaches and to make other evidence-based revisions. These could include contract research organisations (CROs), companies providing ICH-GCP training courses, and groups in the industry working with regulatory processes; all which may benefit at the account of the overly complex and bureaucratic regulations that govern clinical trials today. To facilitate understanding of national requirements, ECRIN-IA developed a comprehensive database (campus.ecrin.org) with information on regulatory and ethical requirements in 22 European countries [55, 56, 75]. This is a starting point, but investigators and sponsors could benefit from additional support from research infrastructures on how to submit to and follow up with authorities in individual countries.

#### Capacity building with research infrastructures

The ECRIN-IA project also included a capacity building programme, fostering the development of local, regional, and national clinical research infrastructures as valuable tools in improving the conduct of investigator-led, single-site or multisite RCTs. These organisations have a key role to play in linking scientific experts and clinical trial professionals with investigators, while providing services to facilitate the development and implementation of national mono-site or multisite trials. Awareness needs to be raised of existing research infrastructures, with efforts to communicate their added value to investigators, disease networks, scientific communities, etc. Moreover, there needs to be greater collaboration between research infrastructures and national networks (of clinical trial units/centres) across borders. This is the mission of ECRIN, which bridges national networks in different European countries, and provides support for trial preparation and management, facilitating multinational collaboration on clinical research in all disease areas. Such multinational collaboration is particularly valuable for certain disease-specific areas as discussed in more detail in other papers on rare diseases, medical devices, and nutrition [11–13]. Disease-specific hurdles include an incomplete understanding of natural history to inform trial design, which in the case of rare diseases is related to low incidence, and thus, poor accrual [11]. For medical devices, a challenge lies in evaluating interventions in a rapid state of flux [12], while nutrition RCTs are particularly challenged by the fact that a true placebo treatment does not exist [13].

The ECRIN-IA project included work packages dedicated to capacity building, supporting the development and upgrade of national clinical research infrastructures to improve Europe’s attractiveness to industry, boost its scientific competitiveness, provide an infrastructure for independent assessments of interventions, and result in better healthcare for European citizens [9, 76, 77]. Currently, through the ECRIN-IA project, ECRIN is expanding its efforts in three areas that will benefit from multinational collaboration: rare diseases, medical devices, and nutrition [11–13].

## Conclusions

Barriers to the conduct of RCTs in general are significant, and it is our hope that by elucidating them, we may help to increase understanding of where and how efforts should be placed. The main barriers identified in the present paper are well-supported by a comprehensive Health Technology Assessment (HTA) report from 1999 [78], which indicates that little change, if any, has occurred during the last two decades. The multinational collaboration on clinical trials has grown considerably during recent years [8, 79]. Analyses show that such collaboration takes time, is costly, and becomes more difficult over increasing distances [8, 79]. It is therefore reassuring that analyses suggest that the best science comes from international collaboration [80]. We propose that multinational collaboration in clinical trial is instrumental in enhancing the conduct of RCTs, especially of academic nature. Governments can draw on the experience of ECRIN-IA to develop context-appropriate solutions to facilitate clinical research and enhance collaboration. Although focused on three areas (medical devices, nutrition, and rare diseases), the tools and activities created through ECRIN-IA are pertinent across all disease areas.

## Additional files

Additional file 1: Literature search strategy. Exact search strategy applied for analyses. (DOCX 13 kb)

Additional file 2: PRISMA 2009 checklist. (DOC 63 kb)

Additional file 3: Relevant references from literature search. Results listed from literature search in the form of relevant publications. (DOCX 28 kb)

## Abbreviations

CDSR: Cochrane Database of Systematic Reviews
CRC: Clinical Research Centre
CTU: Clinical Trial Unit
DARE: Database of Abstracts of Reviews of Effects
ECRIN: European Clinical Research Infrastructure Network
ECRIN-IA: European Clinical Research Infrastructure Network Integrated Activity
EMA: European Medicines Agency
ERA-Net: European Research Area Network
ERare: European Research Area Network for Research Programmes on Rare Diseases
EU FP7: European Union Framework Programme 7
FDA: US Food and Drug Administration
GCP: Good clinical practice
H2020: European Commission Horizon 2020 Research and Innovation programme
HTA: Health Technology Assessment
ICH: International Conference on Harmonization
NHSEED: National Health Service Economic Evaluation Database
PedCRIN: Paediatric Clinical Research Infrastructure Network
RCT: Randomised clinical trial

## Footnotes

1 Funded by the European Union Framework Programme 7 (EU FP7; grant agreement no. 284395), ECRIN-IA involved 23 countries and brought together diverse stakeholders to overcome barriers to clinical research in three particularly difficult areas (rare diseases, medical devices, and nutrition). Specifically, the project aimed to develop tools, services, and infrastructure to facilitate multinational clinical research in Europe, and to support the development of pan-European disease networks to drive clinical projects [7, 81]. This in turn was intended to improve Europe’s attractiveness to industry, boost its scientific competitiveness, and result in better healthcare for European citizens. Originally planned for 4 years (2012–2015), the clinical trials work package was extended until 2017 [7, 81, 76].
