# Supplementary material for: Barriers to the conduct of randomised clinical trials within all disease areas
Source: Trials. 2017 Aug 1;18:360. doi: 10.1186/s13063-017-2099-9 (PMC5539637; doi:10.1186/s13063-017-2099-9)
Supplement: Supplementary file 1 — Literature search strategy. Exact search strategy applied for analyses. (DOCX 13 kb) [file 13063_2017_2099_MOESM1_ESM.docx]

Additional file 1. Literature search strategy

**Barriers to evidence based medicine. C Gluud. Searches performed 17 May 2016**

**Batch-name: 160517_C Gluud_Barriers to EBM**

**Total number identified 113521 references**

**Number of duplicates removed 23028 references**

**Number in list 90493 references**

*The Cochrane Library* (Wiley) (Issue 5 of 12, 2016) (6672 hits – 1358 CDSR, 249 DARE, 4581 CENTRAL, 360 CMR, 45 HTA, 79 NHSEED)

#1 MeSH descriptor: [Evidence-Based Medicine] explode all trees

#2 MeSH descriptor: [Evidence-Based Practice] explode all trees

#3 MeSH descriptor: [Controlled Clinical Trial] explode all trees

#4 MeSH descriptor: [Review] explode all trees

#5 (evidence* and (medicine or practice)) or (clinical trial*) or (systematic review*)

#6 #1 or #2 or #3 or #4 or #5

#7 barrier* or bottle*neck* or obstacle*

#8 #6 and #7

MEDLINE (Ovid SP) (1946 to May 2016) (43779 hits)

1. exp Evidence-Based Medicine/

2. exp Evidence-Based Practice/

3. exp Controlled Clinical Trial/

4. exp "Review"/

5. ((evidence* and (medicine or practice)) or clinical trial* or systematic review*).mp. [mp=title, abstract, original title, name of substance word, subject heading word, keyword heading word, protocol supplementary concept word, rare disease supplementary concept word, unique identifier]

6. 1 or 2 or 3 or 4 or 5

7. (barrier* or bottle*neck* or obstacle*).mp. [mp=title, abstract, original title, name of substance word, subject heading word, keyword heading word, protocol supplementary concept word, rare disease supplementary concept word, unique identifier]

8. 6 and 7

9. limit 8 to (humans and (female or humans or male))

**EMBASE (Ovid SP) (1974 to May 2016) (47725 hits)**

1. exp evidence based practice/

2. controlled study/ or exp controlled clinical trial/

3. ((evidence* and (medicine or practice)) or clinical trial* or systematic review*).mp. [mp=title, abstract, heading word, drug trade name, original title, device manufacturer, drug manufacturer, device trade name, keyword]

4. 1 or 2 or 3

5. (barrier* or bottle*neck* or obstacle*).mp. [mp=title, abstract, heading word, drug trade name, original title, device manufacturer, drug manufacturer, device trade name, keyword]

6. 4 and 5

7. limit 6 to (human and embase)

Science Citation Index Expanded (1900 to May 2016) (15345 hits)

#3 #2 AND #1

#2 TS=(barrier* or bottle*neck* or obstacle*)

#1 TS=((evidence* and (medicine or practice)) or (clinical trial*) or (systematic review*))
