# Supplementary material for: Barriers to the conduct of randomised clinical trials within all disease areas
Source: Trials. 2017 Aug 1;18:360. doi: 10.1186/s13063-017-2099-9 (PMC5539637; doi:10.1186/s13063-017-2099-9)
Supplement: Supplementary file 3 — Relevant references from literature search. Results listed from literature search in the form of relevant publications. (DOCX 28 kb) [file 13063_2017_2099_MOESM3_ESM.docx]

Additional file 3. Relevant references from literature search (n=156)

- European Scienece Foundation. Forward Look - Investigator-Driven Clinical Trials. 2009.
- Anderson G, Boden S, Bridwell K, Ciol M, Deyo R, Dickman C, et al. Breaking down the barriers to restore public confidence: disclosure. Spine. 2002;27(1):6-10.
- Anderson JG. Social, ethical and legal barriers to e-health. International journal of medical informatics. 2007;76(5-6):480-3.
- Areskoug Josefsson K, Kammerlind AS, Sund-Levander M. Evidence-based practice in a multiprofessional context. International Journal of Evidence-based Healthcare. 2012;10(2):117-25.
- Armitage J, Souhami R, Friedman L, Hilbrich L, Holland J, Muhlbaier LH, et al. The impact of privacy and confidentiality laws on the conduct of clinical trials. Clinical Trials (London, England). 2008;5(1):70-4.
- Barrett A, Galvin R, Steinert Y, Scherpbier A, O'Shaughnessy A, Horgan M, et al. A BEME (Best Evidence in Medical Education) systematic review of the use of workplace-based assessment in identifying and remediating poor performance among postgraduate medical trainees. Systems Review. 2015;4:65.
- Bates SE. Phase I clinical trials: overcoming barriers. Clinical Cancer Research. 2010;16(6):1709.
- Beckett M, Quiter E, Ryan G, Berrebi C, Taylor S, Cho M, et al. Bridging the Gap Between Basic Science and Clinical Practice: the Role of Organizations in Addressing Clinician Barriers. Implementation Science. 2011;6.
- Black HL, Priolo C, Akinyemi D, Gonzalez R, Jackson DS, Garcia L, et al. Clearing clinical barriers: enhancing social support using a patient navigator for asthma care. Journal of Asthma. 2010;47(8):913-9.
- Blanco MA, Capello CF, Dorsch JL, Perry G, Zanetti ML. A survey study of evidence-based medicine training in US and Canadian medical schools. Journal of the Medical Library Association. 2014;102(3):160-8.
- Bosse G, Breuer JP, Spies C. The resistance to changing guidelines--what are the challenges and how to meet them. Best Practice & Research. 2006;Clinical Anaesthesiology. 20(3):379-95.
- Boutron I, Estellat C, Guittet L, Dechartres A, Sackett DL, Hrobjartsson A, et al. Methods of blinding in reports of randomized controlled trials assessing pharmacologic treatments: a systematic review. PLoS Medicine / Public Library of Science. 2006;3(10):e425.
- Brousselle A, Lessard C. Economic evaluation to inform health care decision-making: promise, pitfalls and a proposal for an alternative path. Social Science & Medicine. 2011;72(6):832-9.
- Califf RM. Clinical trials bureaucracy: unintended consequences of well-intentioned policy. Clin Trials. 2006;3(6):496-502. Epub 2006/12/16.
- Cameron M, Ray R, Sabesan S. Remote supervision of medical training via videoconference in northern Australia: A qualitative study of the perspectives of supervisors and trainees. BMJ Open. 2015;5(3):1-10.
- Camp JW, Barfield RC, Rodriguez V, Young AJ, Finerman R, Caniza MA. Challenges Faced by Research Ethics Committees in El Salvador: Results From a Focus Group Study. Developing World Bioethics. 2009;9(1):11-7.
- Campbell AJ, Bagley A, Van Heest A, James MA. Challenges of Randomized Controlled Surgical Trials. Orthopedic Clinics of North America. 2010;41(2):145.
- Carrillo JE, Carrillo VA, Perez HR, Salas-Lopez D, Natale-Pereira A, Byron AT. Defining and targeting health care access barriers. Journal of Health Care for the Poor and Underserved. 2011;22(2):562-75.
- Carswell P, Manning B, Long J, Braithwaite J. Building clinical networks: A developmental evaluation framework. BMJ Quality & Safety. 2014;23(5):422-7.
- Castilho V, Almeida R, Miyashiro CAH. Biosimilar trials in developing countries: Detecting and eradicating barriers to patient recruitment. Blood. 2015;Conference: 57th Annual Meeting of the American Society of Hematology, ASH 2015 San Diego, CA United States.
- Cauldwell M, Steer PJ. Regulatory barriers to academically funded randomised controlled trials involving medicines. BJOG. 2015;122(11):1568.
- Chamberlain N. The folly of rewarding silence while hoping for open reporting of adverse medical events - How to realign the rewards. New Zealand Medical Journal. 2008;121(1282):58-66.
- Check DK, Flynn KE, Kramer JM, Weinfurt KP. The use of central irbs for multicenter clinical trials. Clinical Trials (London, England). 2012;Conference: Society for Clinical Trials Annual Meeting 2012 Miami, FL United States.
- Cleary M, Freeman A. Facilitating research within clinical settings: the development of a beginner's guide. International Journal of Mental Health Nursing. 2005;14(3):202-8.
- Cochran C. 'No collaboration, no trial; why collaborator opinion matters'. Trials. 2013;Conference: 2nd Clinical Trials Methodology Conference: Methodology Matters Edinburgh United Kingdom.
- Coleman CH, Ardiot C, Blesson S, Bonnin Y, Bompart F, Colonna P, et al. Improving the Quality of Host Country Ethical Oversight of International Research: the Use of a Collaborative "Pre-Review' Mechanism for a Study of Fexinidazole for Human African Trypanosomiasis. Developing World Bioethics. 2015;15(3):241-7.
- Demotes-Mainard J. Ecrin - New perspective for European clinical research. Basic & Clinical Pharmacology & Toxicology. 2011;Conference: 10th Congress of the European Association for Clinical Pharmacology and Therapeutics Budapest Hungary.
- Demotes-Mainard J, Kubiak C. A European perspective--the European clinical research infrastructures network. Annals of Oncology. 2011;22 Suppl 7:vii44-vii9.
- Duley L, Antman K, Arena J, Avezum A, Blumenthal M, Bosch J, et al. Specific barriers to the conduct of randomized trials. Clinical Trials (London, England). 2008;5(1):40-8.
- Dye C, Bartolomeos K, Moorthy V, Kieny MP. Data sharing in public health emergencies: a call to researchers. Bulletin of the World Health Organization. 2016;94(3):158. Epub 2016/03/12.
- Eapen ZJ, Lauer MS, Temple RJ. The imperative of overcoming barriers to the conduct of large, simple trials. JAMA. 2014;311(14):1397-8.
- Ebell MH. How to find answers to clinical questions. American family physician. 2009;79(4):293-6.
- Eden KB, Totten AM, Kassakian SZ, Gorman PN, McDonagh MS, Devine B, et al. Barriers and Facilitators to Exchanging Health Information: a Systematic Review. International Journal of Medical Informatics. 2016;88:44-51.
- Friedman DB, Kim SH, Tanner A, Bergeron CD, Foster C, General K. How are we communicating about clinical trials?: an assessment of the content and readability of recruitment resources. Contemporary Clinical Trials. 2014;38(2):275-83.
- Gaze B. Privacy and research involving humans. Journal of Law and Medicine. 2003;10(4):410-34.
- Gilbert R, Harron K, Dearden L. Benefits of, and barriers to, reactivating dormant trials. BMJ (Clinical research ed). 2015;351:h5298.
- Gopal RK, Yamashita TE, Prochazka AV. Research without results: inadequate public reporting of clinical trial results. Contemp Clin Trials. 2012;33(3):486-91. Epub 2012/02/22.
- Govindarajan R, Young JW, Harless CL, Hutchins LF. Barriers to clinical trials vary according to the type of trial and the institution. Journal of Clinical Oncology. 2007;25(12):1633-4; au.
- Gul RB, Ali PA. Clinical Trials: the Challenge of Recruitment and Retention of Participants. Journal of Clinical Nursing. 2010;19(1-2):227-33.
- Han D, Trinkaus M, Hogeveen S, Mamdani M, Berry SR, Jang RW, et al. Overcoming obstacles in accessing unfunded oral chemotherapy: Physician experience and challenges. Journal of Oncology Practice. 2013;9(4):188-96.
- Ho K, Jarvis-Selinger S, Borduas F, Frank B, Hall P, Handfield-Jones R, et al. Making interprofessional education work: the strategic roles of the academy.Academic Medicine. 2008;83(10):934-40.
- Holmes BJ, Schellenberg M, Schell K, Scarrow G. How Funding Agencies Can Support Research Use in Healthcare: an Online Province-Wide Survey to Determine Knowledge Translation Training Needs. Implementation Science. 2014;9.
- Holsti M, Adelgais KM, Willis L, Jacobsen K, Clark EB, Byington CL. Developing future clinician scientists and supporting the research infrastructure of an academic medical center. Clinical and Translational Science. 2012;Conference: Translational Science 2012 Meeting Washington, DC United States.
- Hsiehchen D, Espinoza M, Hsieh A. The cooperative landscape of multinational clinical trials. PloS ONE. 2015:10(6).
- Humphreys J. Breaking down the barriers to information sharing. Good Clinical Practice Journal. 2004;11(8):30-1.
- Hyder AA, Corluka A, Winch PJ, El-Shinnawy A, Ghassany H, Malekafzali H, et al. National policy-makers speak out: are researchers giving them what they need? Health Policy and Planning. 2011;26(1):73-82.
- Jelsness-Jorgensen LP. Does a 3-week critical research appraisal course affect how students perceive their appraisal skills and the relevance of research for clinical practice? A repeated cross-sectional survey. Nurse Education Today. 2015;35(1):e1-5.
- Johnson B. Making the link between policy makers, practitioners and research: Awareness, use and perceptions of active living professionals. Journal of Science and Medicine in Sport. 2014;Conference: Be Active 2014 Conference Canberra, ACT Australia.
- Kachalia A, Bates DW. Disclosing medical errors: the view from the USA. Surgeon-Journal of the Royal Colleges of Surgeons of Edinburgh and Ireland. 2014;12(2):64-7.
- Kaplan WA, Wirtz VJ. A research agenda to promote affordable and quality assured medicines. Journal of Pharmaceutical Policy and Practice. 2014:7(1).
- Keinonen T, Keranen T, Klaukka T, Saano V, Ylitalo P, Enlund H. Pharmaceutical Industry's Barriers and Preferences to Conduct Clinical Drug Trials in Finland: a Qualitative Study. European Journal of Pharmaceutical Sciences. 2003;20(1):35-42.
- Keinonen T, Keranen T, Klaukka T, Saano V, Ylitalo P, Enlund H. Investigator Barriers and Preferences to Conduct Clinical Drug Trials in Finland: a Qualitative Study. Pharmacy World and Science. 2003;25(6):251-9.
- Khan MA, Barratt MS, Krugman SD, Serwint JR, Dumont-Driscoll M. Variability of the Institutional Review Board Process Within a National Research Network. Clinical Pediatrics. 2014;53(6):556-60.
- Koshy A, Clark AL. The barriers facing medical research in the UK. British Journal of Cardiology. 2016:23(1).
- Kuehn BM. Clinical Trial Network Removes Barriers Common to Studies of Neurological Diseases. JAMA. 2012;307(7):655.
- Lai TL, Lavori PW, Tsang KW. Adaptive Design of Confirmatory Trials: Advances and Challenges. Contemporary Clinical Trials. 2015;Part A. 45:93-102.
- Laken MA, Egan BM. Overcoming challenges to comparative effectiveness research. Journal of Clinical Hypertension. 2012:Conference: American Society of Hypertension, Inc. 27th Annual Scientific Meeting and Exposition New York, NY United States.
- Lakerveld J, Ijzelenberg W, van Tulder MW, Hellemans IM, Rauwerda JA, van Rossum AC, et al. Motives for (not) participating in a lifestyle intervention trial. BMC Medical Research Methodology. 2008;8:17.
- Lam WWT, Fielding R, Johnston JM, Tin KYK, Leung GM. Identifying Barriers to the Adoption of Evidence-Based Medicine Practice in Clinical Clerks: a Longitudinal Focus Group Study. Medical Education. 2004;38(9):987-97.
- Lang ES, Wyer P, Tabas JA, Krishnan JA. Educational and Research Advances Stemming From the Academic Emergency Medicine Consensus Conference in Knowledge Translation. Academic Emergency Medicine. 2010;17(8):865-9.
- Larsen K, Adamsen L, Bjerregaard L, Madsen JK. There is no gap 'per se' between theory and practice: Research knowledge and clinical knowledge are developed in different contexts and follow their own logic. Nursing Outlook. 2002;50(5):204-12.
- Larson GS, Carey C, Grarup J, Hudson F, Sachi K, Vjecha MJ, et al. Lessons learned: Infrastructure development and financial management for large, publicly funded, international trials. Clin Trials. 2016;13(2):127-36. Epub 2016/02/26.
- Latham J, Murajda L, Forland F, Jansen A. Capacities, Practices and Perceptions of Evidence-Based Public Health in Europe. Euro Surveillance. 2013;18(10):13-7.
- Latifi S, Khalilpour A, Rabiee OL, Amani N. The barriers to research findings utilization among clinical nurses. [Persian]. Journal of Mazandaran University of Medical Sciences. 2012;22(89):87-95.
- Laupacis A. The future of evidence-based medicine. Canadian Journal of Clinical Pharmacology. 2001;8 Suppl A:6A-9A.
- Lauritsen K, Rask-Madsen J. Review: clinical trials in peptic ulcer disease--problems of methodology and interpretation. Alimentary Pharmacology & Therapeutics. 1987;1(2):91-123.
- Lavoie-Tremblay M, Anderson M, Bonneville-Roussy A, Drevniok U, Lavigne GL. Nurse Executives' Perceptions of the Executive Training for Research Application (Extra) Program. Worldviews on evidence-based nursing / Sigma Theta Tau International, Honor Society of Nursing. 2012;9(3):186-92.
- Lawton R, Parker D. Barriers to incident reporting in a healthcare system. Quality & Safety in Health Care. 2002;11(1):15-8.
- Leach MJ. Barriers to conducting randomised controlled trials: lessons learnt from the Horsechestnut & Venous Leg Ulcer Trial (HAVLUT). Contemporary Nurse. 2003;15(1-2):37-47.
- Leach MJ, Gillham D. Are Complementary Medicine Practitioners Implementing Evidence Based Practice? Complementary Therapies in Medicine. 2011;19(3):128-36.
- Ledford CJW, Villagran MM, Kreps GL, Zhao X, McHorney C, Weathers M, et al. "Practicing medicine": Patient perceptions of physician communication and the process of prescription. Patient Education and Counseling. 2010;80(3):384-92.
- Legare F, Boivin A, Van Der Weijden T, Pakenham C, Burgers J, Legare J, et al. Patient and Public Involvement in Clinical Practice Guidelines: a Knowledge Synthesis of Existing Programs. Medical Decision Making. 2011;31(6):E45-E74.
- Leigh JA, Long PW, Barraclough BH. The Clinical Support Systems Program: Supporting System-Wide Improvement. Medical Journal of Australia. 2004;180(10 Suppl):S101-S3.
- Leiter A, Diefenbach MA, Doucette J, Oh WK, Galsky MD. Clinical Trial Awareness: Changes Over Time and Sociodemographic Disparities. Clinical Trials (London, England). 2015;12(3):215-23.
- Leiter A, Sablinski T, Diefenbach M, Foster M, Greenberg A, Holland J, et al. Use of crowdsourcing for cancer clinical trial development. Journal of the National Cancer Institute. 2014:106(10).
- Lengacher CA, Gonzalez LL, Giuliano R, Bennett MP, Cox CE, Reintgen DS. The process of clinical trials: a model for successful clinical trial participation. Oncology Nursing Forum. 2001;28(7):1115-20.
- Leung GM, Johnston JM. Evidence-Based Medical Education - Quo Vadis? Journal of Evaluation in Clinical Practice. 2006;12(3):353-64.
- Lillefjell M, Knudtsen MS, Wist G, Ihlebaek C. From Knowledge to Action in Public Health Management: Experiences From a Norwegian Context. Scandinavian Journal of Public Health. 2013;41(8):771-7.
- Liu L, Leung ELH, Tian XY. Perspective: The clinical trial barriers. Nature. 2011;480(7378 Suppl):S100.
- Llorin-Sangalang J, Verma S, Victor C, Bhatia A, Garcia N. Barriers in Clinical Trial Accrual. European Journal of Cancer. 2014;Conference: 9th European Breast Cancer Conference, EBCC-9 Glasgow United Kingdom.
- Mahlangu J. Research and ethical aspects of clinical trials. Haemophilia. 2012;Conference: 30th International Congress of the World Federation of Hemophilia, Paris, France.
- Mandrekar SJ, Dahlberg SE, Simon R. Improving Clinical Trial Efficiency: Thinking outside the Box. American Society of Clinical Oncology Educational Book. 2015:e141-7.
- Mannava P, Abdullah A, James C, Dodd R, Annear PL. Health systems and noncommunicable diseases in the Asia-Pacific region: a review of the published literature. Asia-Pacific Journal of Public Health. 2015;27(2):NP1-19.
- Manns BJ. Evidence-based decision-making 7: Knowledge translation. Methods in Molecular Biology (Clifton, NJ). 2015;1281:485-500.
- Manolis E, Vamvakas S, Isaac M. New pathway for qualification of novel methodologies in the European Medicines Agency. Proteomics Clinical Applications. 2011;5(5-6):248-55.
- Mansour EG. Barriers to clinical trials. Part III: Knowledge and attitudes of health care providers. Cancer. 1994;74(9 Suppl):2672-5.
- Martinez DA, Tsalatsanis A, Yalcin A, Zayas-Castro JL, Djulbegovic B. Activating clinical trials: A process improvement approach. Trials. 2016:17(1).
- Maynard A, Cookson R. Money or your life? The health-wealth trade-off in pharmaceutical regulation. Journal of Health Services Research & Policy. 2001;6(3):186-9.
- McCabe MS, Varricchio CG, Padberg RM. Efforts to recruit the economically disadvantaged to national clinical trials. Seminars in Oncology Nursing. 1994;10(2):123-9.
- McClung E, Davis S, Chin Kuo M, Lee M, Jeffrey S, Teng N. Overcoming obstacles to clinical trials' enrollment: A lay navigator pilot program focused on Chinese women with cancer. Gynecologic Oncology. 2012;Conference: 2012 Annual Meeting of the Western Association of Gynecologic Oncologists, WAGO 2012 Huntington Beach, CA United States.
- McGoey L. Sequestered Evidence and the Distortion of Clinical Practice Guidelines. Perspectives in Biology and Medicine. 2009;52(2):203-17.
- McGraw D, Greene SM, Miner CS, Staman KL, Welch MJ, Rubel A. Privacy and confidentiality in pragmatic clinical trials. Clin Trials. 2015;12(5):520-9. Epub 2015/09/17.
- McKinney RE, Beskow LM, Ford DE, Lantos JD, McCall J, Patrick-Lake B, et al. Use of Altered Informed Consent in Pragmatic Clinical Research. Clinical Trials (London, England). 2015;12(5):494-502.
- McMahon AD, Conway DI, Macdonald TM, McInnes GT. The unintended consequences of clinical trials regulations. PLoS Medicine. 2009;3(11):e1000131. Epub 2009/11/18.
- McQueen SA, Petrisor B, Bhandari M, Fahim C, McKinnon V, Sonnadara RR. Examining the barriers to meaningful assessment and feedback in medical training. American Journal of Surgery. 2016;211(2):464-75.
- Mentz RJ, Hernandez AF, Berdan LG, Rorick T, O'Brien EC, Ibarra JC, et al. Good Clinical Practice Guidance and Pragmatic Clinical Trials: Balancing the Best of Both Worlds. Circulation. 2016;133(9):872-80. Epub 2016/03/02.
- Merlo G, Page K, Ratcliffe J, Halton K, Graves N. Bridging the gap: exploring the barriers to using economic evidence in healthcare decision making and strategies for improving uptake. Applied Health Economics and Health Policy. 2015;13(3):303-9.
- Miller TJ, Chaitt DG, Kopka SL, Albert SM, Young KG, Sevastita VS, et al. Transforming research barriers into process change through the protocol development program. Clinical and Translational Science. 2013;Conference: Translational Science 2013 Meeting Washington, DC United States.
- Mills N, Blazeby J, Hamdy F, Neal D, Campbell B, Donovan J. Recruiters to randomised trials can be trained to facilitate recruitment and informed consent by exploring patients' treatment preferences. Trials. 2013;Conference: 2nd Clinical Trials Methodology Conference: Methodology Matters Edinburgh United Kingdom.
- Mills N, Blazeby JM, Hamdy FC, Neal DE, Campbell B, Wilson C, et al. Training Recruiters to Randomized Trials to Facilitate Recruitment and Informed Consent by Exploring Patients' Treatment Preferences. Trials. 2014;15.
- Mirbaha F, Shalviri G, Yazdizadeh B, Gholami K, Majdzadeh R. Perceived Barriers to Reporting Adverse Drug Events in Hospitals: a Qualitative Study Using Theoretical Domains Framework Approach. Implementation Science. 2015;10.
- Montagne M. Failure to consider a radically new scientific idea or theory. Substance Use & Misuse. 2012;47(13-14):1469-72.
- Morrison J, Pons-Vigues M, Becares L, Burstrom B, Gandarillas A, Dominguez-Berjon F, et al. Health Inequalities in European Cities: Perceptions and Beliefs Among Local Policymakers. BMJ Open. 2014;4(5).
- Mosconi P, Roberto A. Open-Access Clinical Trial Registries: the Italian Scenario. Trials. 2012;13.
- Murphy JFA. Clinical networks. Irish Medical Journal. 2013;106(3):1.
- Nair SC, Ibrahim H, Celentano DD. Clinical Trials in the Middle East and North Africa (Mena) Region: Grandstanding or Grandeur? Contemporary Clinical Trials. 2013;36(2):704-10.
- Ness RB. Invited Commentary: Population-Based Human Subjects Research in the Era of Enhanced Privacy Regulation. American Journal of Epidemiology. 2010;172(6):648-50.
- Noble DJ, Pronovost PJ. Underreporting of patient safety incidents reduces health care's ability to quantify and accurately measure harm reduction. Journal of Patient Safety. 2010;6(4):247-50.
- Oliver K, Innvar S, Lorenc T, Woodman J, Thomas J. A systematic review of barriers to and facilitators of the use of evidence by policymakers. BMC Health Services Research. 2014;14:2.
- Oliveri RS, Gluud C, Wille-Jorgensen PA. Hospital Doctors' Self-Rated Skills in and Use of Evidence-Based Medicine - a Questionnaire Survey. Journal of Evaluation in Clinical Practice. 2004;10(2):219-26.
- Orloff JJ, Stanski D. Innovative approaches to clinical development and trial design. Annali dell'Istituto Superiore di Sanita. 2011;47(1):8-13.
- Orton L, Lloyd-Williams F, Taylor-Robinson D, O'Flaherty M, Capewell S. The use of research evidence in public health decision making processes: systematic review. PLoS ONE. 2011;6(7):e21704.
- Page SJ, Persch AC. Recruitment, retention, and blinding in clinical trials. American Journal of Occupational Therapy. 2013;67(2):154-61.
- Parker C, Weiner M, Reeves M. Health Information Exchanges-Unfulfilled Promise as a Data Source for Clinical Research. International Journal of Medical Informatics. 2016;87:1-9.
- Pearce W, Raman S, Turner A. Randomised trials in context: practical problems and social aspects of evidence-based medicine and policy. Trials. 2015;16:394. Epub 2015/09/06.
- Pelusio RM, Herr BE. Removing the barriers to performing clinical trials in academic settings. Good Clinical Practice Journal. 1998;5(2):17-9.
- Penataro JS, Ezzeldin M, Sanz N, Calvo G, Carne X, Kubiak C. ECRIN (European clinical research infrastructure network): The added value of interoperability and Pan-European interconnection on clinical research. Clinical Therapeutics. 2015;Conference: 12th Congress of the European Association for Clinical Pharmacology and Therapeutics, EACPT 2015 Madrid Spain.
- Plumb M, Price W, Kavanaugh-Lynch MHE. Funding community-based participatory research: Lessons learned. Journal of Interprofessional Care. 2004;18(4):428-39.
- Poorolajal J, Rezaie S, Aghighi N. Barriers to medical error reporting. International Journal of Preventive Medicine. 2015:2015-October.
- Prescott RJ, Civil I. Lies, damn lies and statistics: errors and omission in papers submitted to INJURY 2010-2012. Injury. 2013;44(1):6-11. Epub 2012/11/28.
- Prescott RJ, Counsell CE, Gillespie WJ, Grant AM, Russell IT, Kiauka S, et al. Factors that limit the quality, number and progress of randomised controlled trials. Health Technology Assessment. 1999;3(20):iii-139.
- Raftery J, Kerr C, Hawker S, Powell J. Paying clinicians to join clinical trials: a review of guidelines and interview study of trialists. Trials. 2009;10:15.
- Rengerink KO, Opmeer BC, Logtenberg SLM, Hooft L, Bloemenkamp KWM, Haak MC, et al. Improving Participation of Patients in Clinical Trials - Rationale and Design of Impact. BMC Medical Research Methodology. 2010;10.
- Reveiz L, Krleza-Jeric K, Chan AW, Aguiar S. Do trialists endorse clinical trial registration? Survey of a PubMed sample [abstract]. XIV Cochrane Colloquium; 2006 October 23-26; Dublin, Ireland. 2006:63.
- Robinson L, Newton J, Dawson P. Professionals and the public: Power or partnership in health research? Journal of Evaluation in Clinical Practice. 2012;18(2):276-82.
- Ross S, Grant A, Counsell C, Gillespie W, Russell I, Prescott R. Barriers to participation in randomised controlled trials: a systematic review. Journal of Clinical Epidemiology. 1999;52(12):1143-56.
- Ross S, Magee L, Wood S. Open versus closed access to full academic trial protocols: Advantages and disadvantages. Clinical Trials (London, England). 2011;Conference: 32nd Meeting of the Society of Clinical Trials Vancouver, BC Canada.
- Rowland J. Securing funding for your research. Psycho-Oncology. 2015;Conference: 2015 World Congress of Psycho-Oncology Washington, DC United States.
- Sanson-Fisher R, Brand M, Shakeshaft A, Haber P, Day C, Conigrave K, et al. Forming a national multicentre collaboration to conduct clinical trials: increasing high-quality research in the drug and alcohol field. Drug and Alcohol Review. 2010;29(5):469-74.
- Schain WS. Barriers to clinical trials. Part II: Knowledge and attitudes of potential participants. Cancer. 1994;74(9 Suppl):2666-71.
- Shannon J. Barriers to Access of Clinical Data Glaxosmithkline on Its Remote Desktop Interface for Independent Reanalysis of Drug Trial Data. BMJ. 2014;349.
- Shore BJ, Nasreddine AY, Kocher MS. Overcoming the funding challenge: the cost of randomized controlled trials in the next decade. Journal of Bone & Joint Surgery - American Volume. 2012;94 Suppl 1:101-6.
- Sinclair DG. Evidence over barriers: important but not enough. HealthcarePapers. 2011;11(2):57-60; discussion 4-7.
- Soreide K, Alderson D, Bergenfelz A, Beynon J, Connor S, Deckelbaum DL, et al. Strategies to improve clinical research in surgery through international collaboration. Lancet. 2013;382(9898):1140-51.
- Tenopir C, Allard S, Douglass K, Aydinoglu AU, Wu L, Read E, et al. Data sharing by scientists: Practices and perceptions. PloS ONE. 2011:6 () (no pagination.
- Tragus R, Cody JD. An Evaluation Tool for Collaborative Clinical Research Centers. Cts-Clinical and Translational Science. 2013;6(3):244-7.
- Tricco AC, Cardoso R, Thomas SM, Motiwala S, Sullivan S, Kealey MR, et al. Barriers and Facilitators to Uptake of Systematic Reviews by Policy Makers and Health Care Managers: a Scoping Review. Implementation Science. 2016;11.
- Ubbink DT, Guyatt GH, Vermeulen H. Framework of Policy Recommendations for Implementation of Evidence-Based Practice: a Systematic Scoping Review. BMJ Open. 2013;3(1).
- van Panhuis WG, Paul P, Emerson C, Grefenstette J, Wilder R, Herbst AJ, et al. A systematic review of barriers to data sharing in public health. BMC Public Health. 2014;14:1144.
- Vedelo TW, Lomborg K. Reported challenges in nurse-led randomised controlled trials: an integrative review of the literature. Scandinavian Journal of Caring Sciences. 2011;25(1):194-200.
- Vickers AJ. Clinical Trials in Crisis: Four Simple Methodologic Fixes. Clinical Trials (London, England). 2014;11(6):615-21.
- Virk KP. Addressing issues affecting clinical trials in Brazil. Clinical Research and Regulatory Affairs. 2010;27(2):52-9.
- Wagle N, Goldman DP, Kilgore M. Re: Surveys identify barriers to participation in clinical trials [2]. Journal of the National Cancer Institute. 2001;93(3):238-9.
- Wahabi HA, Siddiqui AR, Mohamed AG, Al-Hazmi AM, Zakaria N, Al-Ansary LA. Evidence-Based Decision Making in Public Health: Capacity Building for Public Health Students at King Saud University in Riyadh. BioMed Research International. 2015:2015.
- Wallace J, Byrne C, Clarke M. Improving the uptake of systematic reviews: a systematic review of intervention effectiveness and relevance. BMJ Open. 2014;4(10):e005834.
- Wallace J, Nwosu B, Clarke M. Barriers to the uptake of evidence from systematic reviews and meta-analyses: A systematic review of decision makers' perceptions. BMJ Open. 2012:2(5).
- Wang-Gillam A, Novello S, Williams K, Gao F, Scagliotti GV, Govindan R. An international comparison of barriers in opening oncology clinical trials [abstract]. Journal of Clinical Oncology Supplement: 2009 Annual Meeting Proceedings Part I. 2009;27(15S):342s.
- Waqa G, Mavoa H, Snowdon W, Moodie M, Nadakuitavuki R, Mc Cabe M, et al. Participants' perceptions of a knowledge-brokering strategy to facilitate evidence-informed policy-making in Fiji. BMC Public Health. 2013;13:725.
- Weckstein DJ, Thomas CA, Emery IF, Shea BF, Fleury A, White ME, et al. Assessment of perceived cost to the patient and other barriers to clinical trial participation. Journal of Oncology Practice. 2011;7(5):330-3.
- Weitman SD, Ramirez AG, Chalela P, Munoz E, Pollock BH. Overcoming the Barriers to Early-Phase Clinical Trials (Epct): Increasing Epct Minority Accrual. Journal of Clinical Oncology. 2012;30(15).
- Woodrow R, Jacobs A, Llewellyn P, Magrann J, Eastmond N. Publication of past and future clinical trial data: Perspectives and opinions from a survey of 607 medical publication professionals. Current Medical Research and Opinion. 2012;Conference: 8th Annual Meeting of the International Society for Medical Publication Professionals, ISMPP: 'Practical Solutions for a Complex Medical Publications World' Cheshire United Kingdom.
- Woods K. Debate 'European Clinical Trials are grossly over-regulated and under-supported' - The case against. Basic & Clinical Pharmacology & Toxicology. 2009;Conference: 9th Congress of the European Association for Clinical Pharmacology and Therapeutics Edinburgh United Kingdom.
- Wright JG, Katz JN, Losina E. Clinical trials in orthopaedics research. Part I. Cultural and practical barriers to randomized trials in orthopaedics. Journal of Bone & Joint Surgery - American Volume. 2011;93(5):e15.
- Yarbrough AK, Smith TB. Technology acceptance among physicians: a new take on TAM. Medical Care Research and Review. 2007;64(6):650-72.
- Yoruk S, Tetik E, Karaalp A. Reasons for non-participation of Turkish patients in international clinical trials. Marmara Medical Journal. 2012;25(2):78-82.
- Zardo P, Collie A, Livingstone C. External factors affecting decision-making and use of evidence in an Australian public health policy environment. Social Science & Medicine. 2014;108:120-7.
